# Effectiveness of a Social Cognitive Theory-based health education intervention on depression (SCODESS) among mothers of children with cancer in Klang Valley, Selangor, Malaysia—A quasi-experimental study

**DOI:** 10.1371/journal.pone.0318104

**Published:** 2025-02-05

**Authors:** Wan Syahirah Wan Ghazali, Halimatus Sakdiah Minhat, Sherina Mohd Sidik, Iqmer Nashriq Mohd Nazan

**Affiliations:** 1 Department of Community Health, Faculty of Medicine and Health Sciences, Universiti Putra Malaysia, Seri Kembangan, Malaysia; 2 Department of Psychiatry, Faculty of Medicine, and Health Sciences, Universiti Putra Malaysia, Seri Kembangan, Malaysia; Universiti Monash Malaysia: Monash University Malaysia, MALAYSIA

## Abstract

**Background:**

Mothers caring for children with cancer often experience depression, affecting maternal and family well-being. Prior studies suggest that theory-based health education can significantly reduce this depression.

**Objectives:**

This study aimed to develop, validate, implement, and evaluate the effects of a Social Cognitive Theory (SCT)-based health education intervention on depression (SCODESS), as well as cancer-related knowledge, self-efficacy, perceived stress, coping skills, and social support among mothers of children with cancer at University Hospitals in Klang Valley.

**Methods:**

A quasi-experimental study was conducted with mothers from two University Hospitals in Klang Valley, Selangor. The intervention group is Hospital Pakar Kanak-Kanak Universiti Kebangsaan Malaysia (HPKK UKM) and the control group is the Universiti Malaya Medical Centre (UMMC). A total of 95 participants were included (50 intervention, 45 control). The intervention comprised online health education videos delivered over one week, and the control group received a poster. Data were collected at baseline (T1), immediately post-intervention (T2), and at two months post-intervention (T3). The effects of SCODESS intervention were analysed using Generalised Estimating Equation (GEE) analysis.

**Results:**

The baseline response rate was 60.53% with a 2.17% loss to follow-up at T2 and 7.60% at T3. The GEE analysis showed no significant effects of SCODESS intervention on depression scores at T2 (p = 0.909) and T3 (p = 0.622) compared to the control group at baseline. However, statistically significant increases were observed in cancer-related knowledge scores at T2 (β = 0.66, 95%CI: 0.21, 9.20, p = 0.002) and T3 (β = 1.18, 95%CI: 0.65, 1.70, p<0.001), and in the problem-focused coping scores at T2 (*β* = 2.50, 95% CI 0.42, 4.58, p-value = 0.018), and T3 (*β* = 2.42, 95% CI 0.13, 4.72, p-value = 0.038) in the intervention group compared to the control group at baseline. No significant intervention effects were observed on other outcomes.

**Discussion:**

This study validated the applicability of SCT-based intervention on depression scores among mothers of children with cancer. The SCODESS intervention did not significantly reduce depression scores but significantly increased cancer-related knowledge and problem-focused coping scores. As a potential preventive strategy for depression, the content of the SCODESS intervention should be revisited, emphasizing cancer-related knowledge and problem-focused coping as crucial components. It is recommended that tailored interventions focusing on these areas be offered to every mother of children with cancer, whether they are in the ward, clinic, or daycare.

## Introduction

The Malaysia National Cancer Registry (MNCR) (2021) and the National Cancer Institute, United States (2020), classified childhood cancer as cancer that occurs between birth and 14 years of age. However, certain types of childhood cancers peaked in the young adolescent group which is between the ages of 15 to 19 years old. Therefore, some literature classified cancers that occur among these young adolescent age groups as childhood cancers [1]. Globally, the WHO estimates that 400,000 children and adolescents aged 0 to 19 years develop cancer annually [2]. In Malaysia, the Malaysia National Cancer Registry (2021) reported an age-standardized rate of 3.81 per 100,000 males aged 0 to 19 years and 3.07 per 100,000 females aged 0 to 19 years for cancers diagnosed between 2012 and 2016. The incidence of childhood cancers in Malaysia increased from 4089 cases between 2007 and 2011 to 4303 cases between 2012 and 2016 [3].

Childhood cancers remain among the most impactful chronic diseases affecting children. The impacts of childhood cancer extend not only to the child but also to family members, relatives, and communities [4]. Childhood cancer acts as the turning point for the changes in the family dynamic which may be affected by frequent hospital follow-up, intensive treatment, and marital and financial challenges. Furthermore, the constant fear of relapse, death, or the uncertainties that lie ahead greatly affects the whole family’s perspective on dreams, hope, faith, life, and death to the extent that childhood cancer can be considered a family disease [4, 5].

One of the impacts of learning that the child has a life-threatening diagnosis reverberates through the mental health of the parents, with depression being one of the most common mental health issues reported by parents. The American Psychological Association (APA) defined depression as a negative affective state, ranging from unhappiness and discontent to an extreme feeling of sadness, pessimism, and despondency, that interferes with daily life [6]. Symptoms of depression include feeling sad or depressed mood, loss of interests or activities once enjoyed, changes in appetite, trouble sleeping or sleeping too much, loss of energy or increased fatigue, feeling worthless or guilty, difficulty thinking, and concentrating, and thoughts of death or suicide [6]. Findings from a meta-analysis on the prevalence of mental illnesses among parents of children with cancer showed that the pooled prevalence of depression was 28%, anxiety was 21% and Post-Traumatic Stress Disorder (PTSD) was 26% [7].

Among parents of children with cancer, studies have shown that mothers are more vulnerable to symptoms of depression than fathers [8–13]. In Basra, Iraq, depression was reported in 77.2% of mothers and 57.1% of fathers of children with cancer [10]. In Turkey, 36.4% of the mothers and 25.0% of the fathers had severe symptoms of depression, 18.2% of mothers and 4.5% of fathers had moderate symptoms of depression [8]. Among mothers, the prevalence of depression among these mothers varies between countries and the severity of the symptoms. In Saudi Arabia, 47.8% of the mothers had mild depression, 20.3% had moderate symptoms, 5.8% had moderately severe symptoms and 5.8% had severe symptoms of depression [14]. In Turkey, 18.2% of the mothers had moderate depression and 36.4% had severe depression [8]. Meanwhile, in Karachi, 69.0% of the mothers had mild depression, 25.0% had moderate, 5.0% had severe depression and 1.0% had very severe depression [15].

Several factors are associated with depression among mothers of children with cancer as reported by previous literature [16]. Younger mothers [17], being single [18] lower family income or socioeconomic condition [17, 19] and lower educational level [17–19]; were reported as having higher risks of developing depression compared to otherwise. Additionally, mothers of children with cancer who experienced higher stress [18, 20] as well as poor social support and certain coping skills also have a higher risk of depression [13]. Furthermore, a higher sense of self-efficacy among parents of children with cancer may protect against adverse effects of caregiver burden such as depression [21]. Apart from that, parents with higher cancer knowledge reported less stress as they had more engagement and involvement with their child’s ongoing treatment [22]. The involvement of the parents as part of the treating team helps to alleviate uncertainties, fear, and misunderstanding regarding the complexity of the treatment, subsequently helping to reduce stress or depression among the parents.

Evidence suggests intervention programs can improve mental health outcomes among parents of children with cancer. Globally, one of the frequently implemented interventions among family caretakers in oncology is health education intervention that is delivered in various modalities. The content of the intervention varies depending on the specific study objectives and targeted population [23, 24]. Regardless, findings of the intervention studies showed significant improvement in mental health including depression of the mothers after the implementation of the intervention. Additionally, several studies have applied Social Cognitive Theory (SCT) as the theoretical framework for intervention studies involving caretakers of chronic illnesses [25–27]. The SCT theory was developed by Bandura in 1989 and explains human behavior in a three-way reciprocal model (personal, behavior, environment) that continually interacts with each other [27]. The solid framework of this theory provides a more structured and comprehensive intervention to address depression among mothers of children with cancer. Thus, the objective of this SCODESS study is to develop, validate, implement, and evaluate the effectiveness of Social Cognitive Theory-based health education intervention on depression, cancer-related knowledge, caretaker self-efficacy, perceived stress, coping skills, perceived social support, and depression among mothers of children with cancer at University Hospitals in Klang Valley.

## Material and methods

### Study design

This study is a two-arm quasi-experimental study design with intervention and control groups. Before commencing recruitment, this study was registered with the Thai Clinical Trials Registry (TCTR20230410006). It was conducted according to the guidelines of the Declaration of Helsinki, with informed consent obtained from each participant. Ethical approval was obtained from the Ethics Committee at Universiti Putra Malaysia (JKEUPM-2018-054) Universiti Kebangsaan Malaysia (UKM PPI/111/8/JEP-2023-032), Universiti Malaya (20221213–11798) dan Universiti Sains Malaysia (USM/JEPEM/22120760). This study involved only two university teaching hospitals at Klang Valley: Universiti Kebangsaan Malaysia Specialist Children Hospital (UKM HPKK) and University Malaya Medical Centre (UMMC). As a quasi-experimental study does not rely on random assignment, UKM HPKK was designated as the intervention hospital and UMMC as the control hospital.

This study consists of a screening phase and an intervention phase. In the screening phase, mothers with mild to severe depression were identified using Patient Health Questionnaires (PHQ-9). In the intervention phase, the effectiveness of the SCODESS intervention was assessed among eligible participants. In every phase, each participant was informed regarding the study by the researchers. Verbal and written informed consent was taken from the eligible respondents who agreed to participate in the study. Participants were recruited from the outpatient clinic, daycare, and inpatient units at both UKM HPKK and UMMC. Participants completed the first set of questionnaires at baseline (T1), immediately post-intervention (T2), and at 2 months post-intervention (T3). The intervention lasted for one week (Day 1 to Day 7). The whole process of data collection occurred from 3^rd^ April 2023 until 2^nd^ October 2023.

### Participants

**Inclusion criteria.** Participants were eligible if they were Malaysian mothers of children with cancer aged between 0–18 years, who receiving cancer treatment at University Hospitals at Klang Valley. Participants must score ≥ 5 on the Patient Health Questionnaire (PHQ-9), which corresponds with mild to higher depression symptoms. Participants must understand Malay or English language. **Exclusion criteria.** Participants were ineligible if they were not the guardians of a child with cancer, and if they had a history of clinical depression.

### Intervention

#### Development and delivery of online Social Cognitive Theory-based health education intervention on depression (SCODESS) among mothers of children with cancer

The SCODESS module was newly developed by the researcher based on an extensive literature review and incorporating various inputs from experts in the field. This study uses the ADDIE model as a guide for the systematic development of the module. The ADDIE model, which stands for Analysis, Design, Development, Implement, and Evaluation is a well-established instructional design model used by many researchers [28]. Each phase was meticulously executed to ensure the module’s relevance, accuracy, and effectiveness in addressing depression among mothers of children with cancer. The development of the SCODESS module using the ADDIE model as a guide is shown in Table 1.

**Table 1:** SCODESS module development.

Phase	Module development and content
Analysis	Determine the need to develop the module by an extensive literature review.
	Engaged with informal discussion among mothers of children with cancer on the need to address their mental health
Design	Identify the objectives, research hypothesis
	Design the module with advice from the experts
Development	Development of the module
	Discussion with the experts
Implementation	Face validity of the module to pilot study participants
Evaluation	Evaluation of the findings and feedback for the module improvement

Based on an extensive literature review, the researcher identified Social Cognitive Theory (SCT) which consists of personal, behavioral, and environmental constructs that fit nicely with the factors associated with depression among mothers [16]. Moreover, SCT-based psychoeducational interventions have been applied among caretakers of cancer patients [27], caretakers of chronic kidney disease [25], and depressed female adolescents [29]. Therefore, SCT was chosen as the theoretical framework for the SCODESS intervention, which includes ‘Personal’ constructs (*Childhood Cancer Related Knowledge (Module 1)*, *Depression among Mothers of Children with Cancer (Module 2)*, *Perceived Stress (Module 3)*, and *Caretaker Self Efficacy (Module 4)*. Subsequently, Behavioral Constructs concentrated on *coping skills (Module 5)*, and Environmental Constructs identified *Perceived social support (Module 6)*. Table 2 shows the application of SCT as the framework in the health education module.

**Table 2:** The application of SCT construct in the SCODESS module.

Module	Content	Theory Construct
1.	Childhood cancer-related knowledge	Personal
2.	Depression among mothers of children with cancer,	Personal
3.	Perceived Stress	Personal
4.	Caretaker Self Efficacy	Personal
5.	Coping Skills	Behavior
6.	Perceived Social Support.	Environment

The SCODESS intervention was developed into a series of health education videos and delivered online to the respondents. During the intervention phase, participants were allocated to WhatsApp Group 1 until WhatsApp Group 5 depending on the list name of the participants. Each WhatsApp group consisted of a maximum of 10 respondents. Subsequently, videos from each module were delivered via the WhatsApp group on alternate days (Day 1, Day 3, and Day 5) to give more time for the respondents to go through each video presentation. The post-intervention questionnaire was distributed on Day 7 of the intervention (T2). After two months, respondents were contacted for the 2-month post-intervention survey (T3).

The control group in this study received posters on depression, the hotline number, and the link for deep breathing exercises and relaxation techniques. The same videos and interventions were distributed once the intervention and control group completed their final questionnaire, completing the waitlist procedure. Table 3 shows the summary of the SCODESS intervention content and module delivery.

**Table 3:** Summary of the SCODESS intervention content and module delivery.

Module	SCT Construct	Content	Format of delivery	Estimated time (minutes)	Day
Baseline survey		Questionnaire	Manual form / online Google form	20	Pre-intervention
Module 1	Personal	Depression among mothers of children with cancer (definition, signs and symptoms, why mothers are more vulnerable to depression, management)	Recorded lecture with PowerPoint presentation	5	Day 1
Module 2	Personal	Basic knowledge of childhood cancer (Definition, signs and symptoms, diagnosis, treatment)	Recorded lecture with PowerPoint presentation	5	Day 1
Module 3	Personal	Perceived Stress (definition of stress, video presentation on breathing and relaxation technique, practical session)	Video presentation	5	Day 3
Module 4	Personal	Caretaker self-efficacy (definition, factors contribute to high self-efficacy)	Recorded lecture with PowerPoint presentation.	10	Day 3
		Video of sharing session with a mother of a child with cancer (resilience, emotional connectivity)	Recorded video of sharing sessions x 2		
Module 5	Behavior	Coping skills (Emphasize problem-solving skills, discussion)	Video presentation	5	Day 5
Module 6	Environment	Perceived social support (definition, barriers to social support, ways to increase social support)	Video presentation.	5	Day 5
		Importance of social support from the mother’s point of view			
Post-intervention i) survey 1	-	Questionnaire	Google form link	20	Day 7
ii) survey 2					2-months post-intervention

### Assessment and outcomes

#### Primary outcome

**Depression** was measured using the Patient Health Questionnaire-9 (PHQ-9). The PHQ-9 is a 9 items self-administered questionnaire based on DSM-IV criteria for major depression [30]. The questionnaire consists of nine items referring to the symptoms experienced by the participants two weeks before answering the questionnaires [31]. The scores range from 0 to 27, as each item scored from 0 (not at all), 1 (several days), 2 (More than half the days), and 3 (nearly every day). Higher scores indicated higher depression levels. The PHQ-9 scores can be categorized into 5, 10, 15, and 20 representing mild, moderate, moderately severe, and severe depression [31]. The Cronbach Alpha for the PHQ-9 questionnaire in this study was 0.844.

#### Secondary outcomes

**Cancer-related knowledge** was measured using 10 items developed based on literature reviews and a previous study by Othman et. al (2012) [22]. It consists of ten RIGHT/WRONG/UNSURE items about childhood cancer and its management and comprises questionnaires related to the definition, type, causes, symptoms, treatment modalities, management, and side effects [22]. Only the correct answer scored 1 and the incorrect/unsure answer scored 0. Higher scores indicated greater cancer knowledge.

**Perceived stress** was measured using an adapted 8-item Modified Perceived Stress Scale. The initial internal consistency reliability analysis of the 10-item questionnaire yielded a Cronbach Alpha of 0.515, indicating a moderate internal consistency. To improve the reliability, further item analysis was conducted. Two items were identified as contributing to the low overall reliability score. After removing the two items, the revised Cronbach Alpha value was 0.739, indicating improvement in the internal consistency. This value indicates a high-reliability value; suggesting the modified perceived stress scale is reliable for measuring the intended outcome. The Likert scale ranging from 0 (never) to 4 (very often) measures the extent to which one appraises events during the past month as stressful by Cohen et. al., (1983) [32]. Higher scores indicated higher perceived stress.

**Caretaker self-efficacy** was measured using the Modified Caretaker Self-Efficacy Scale (CaSES) [28]. The initial questionnaires included 21 items ranging from 0 (not at all confident) to 3 (very confident). However, during the content validation process of the questionnaire, three panels agreed to remove *Item 5*: *I can help my child to make decisions about cancer treatment*. The decision was made as the item was found to be irrelevant in the context of pediatric cancer, where the healthcare professional and parents made decisions rather than the children themselves. The Cronbach Alpha for the 20-item questionnaire was 0.947. The total score ranged between 0 to 63 with higher scores indicating greater caretaker self-efficacy. The caretaker self-efficacy can further be categorized into a low level of caretaker self-efficacy(score 0–20), a moderate level of caretaker self-efficacy (score 21–41), and a high level of caretaker self-efficacy (score 42–63) [33].

**Coping skills** were measured using a Modified Brief COPE questionnaire on a 4-Likert Scale, from 1 (haven’t been doing this at all) 2 (a little bit), 3 (a medium amount), and 4 (I’ve been doing this a lot). The original Brief COPE questionnaire consisted of 28 Likert Scale items. However, based on the face and content validity assessment, 11 items were suggested for removal, leaving 17 items in total. The items were removed due to redundancy and the need to create a concise and relevant questionnaire for depressed mothers. The Cronbach Alpha for the 17-item questionnaire was 0.890. The coping skills were divided into two categories which were ‘Problem Focused Coping’ and ‘Emotion Focused Coping’. Higher scores indicated higher coping skills either Problem Focused or Emotion-Focused.

**Social support** was measured using a Multidimensional Scale of Perceived Social Support (MSPSS-M) questionnaire which consisted of 12 items on a 7 Likert Scale, from 1 (very strongly disagree) to 7 (very strongly agree) [34]. The scale was categorized into subscales of significant other, family, and friends. Based on the mean score, the scale can be divided into low support (score 1.0 to 2.9), moderate support (score 3.0 to 5), and high support (score 5.1 to 9) [34]. The Cronbach Alpha for this questionnaire was 1.000.

### Statistical analysis

Data was analysed using IBM SPSS Version 25. A descriptive statistical analysis of participants’ sociodemographic characteristics, baseline depression scores, cancer-related knowledge scores, perceived stress scores, caretaker self-efficacy scores, coping skills scores, and social support scores was presented as mean and standard deviation for normally distributed data and as median and interquartile range for not normally distributed data. Categorical variables were presented as frequency and percentage.

Univariate statistical analysis was conducted to compare the baseline between the intervention and control groups. For continuous and normally distributed data, an independent t-test was used to compare the means of two groups, whereas, for continuous data that were not normally distributed, the Mann-Whitney test was used to compare the median between the two groups. The Chi-square and Fisher’s Exact Test were used to compare the differences in categorical data. Subsequently, Generalised Equation E (GEE) was used in this study to determine the effectiveness of the theory-based health education intervention to reduce depression among mothers of children with cancer.

Generalized Estimating Equations (GEE) is a statistical technique used to analyze correlated data, such as repeated measures, where observations within the same subject or group are not independent. It provides robust standard errors that account for intra-subject correlation, making it particularly useful for longitudinal or clustered data. This method is commonly used in health research involving repeated measures or longitudinal data [35]. The level of significance is at a p-value of 0.05 with a confidence interval of 95%. If p<0.05, the null hypothesis is rejected.

### Ethical issues

Ethical approval was obtained from the Ethics Committees of Universiti Putra Malaysia (JKEUPM-2018-054) Universiti Kebangsaan Malaysia (UKM PPI/111/8/JEP-2023-032), Universiti Malaya (20221213–11798) and Universiti Sains Malaysia (USM/JEPEM/22120760. The study protocol was registered with the Thai Clinical Trials Registry (TCTR20230410006). Privacy and confidentiality were maintained, with all data stored on a password-protected encrypted computer. Participants could withdraw at any time without penalty.

Mothers identified as being in a serious state of self-harm (item 9, score 3) on the PHQ-9 were contacted and offered referral to a Counsellor at the nearest health clinics. If they declined, they were advised to seek help if symptoms worsened and to visit the nearest health clinic if they experienced thoughts of self-harm. The Mental Health Hotline number was also provided. At the end of the study, mothers scoring 10 or higher on the PHQ-9 screening were referred to the nearest health clinics for further assessment and management of their depression, with their consent.

## Results

### Response rate

**Fig 1:**
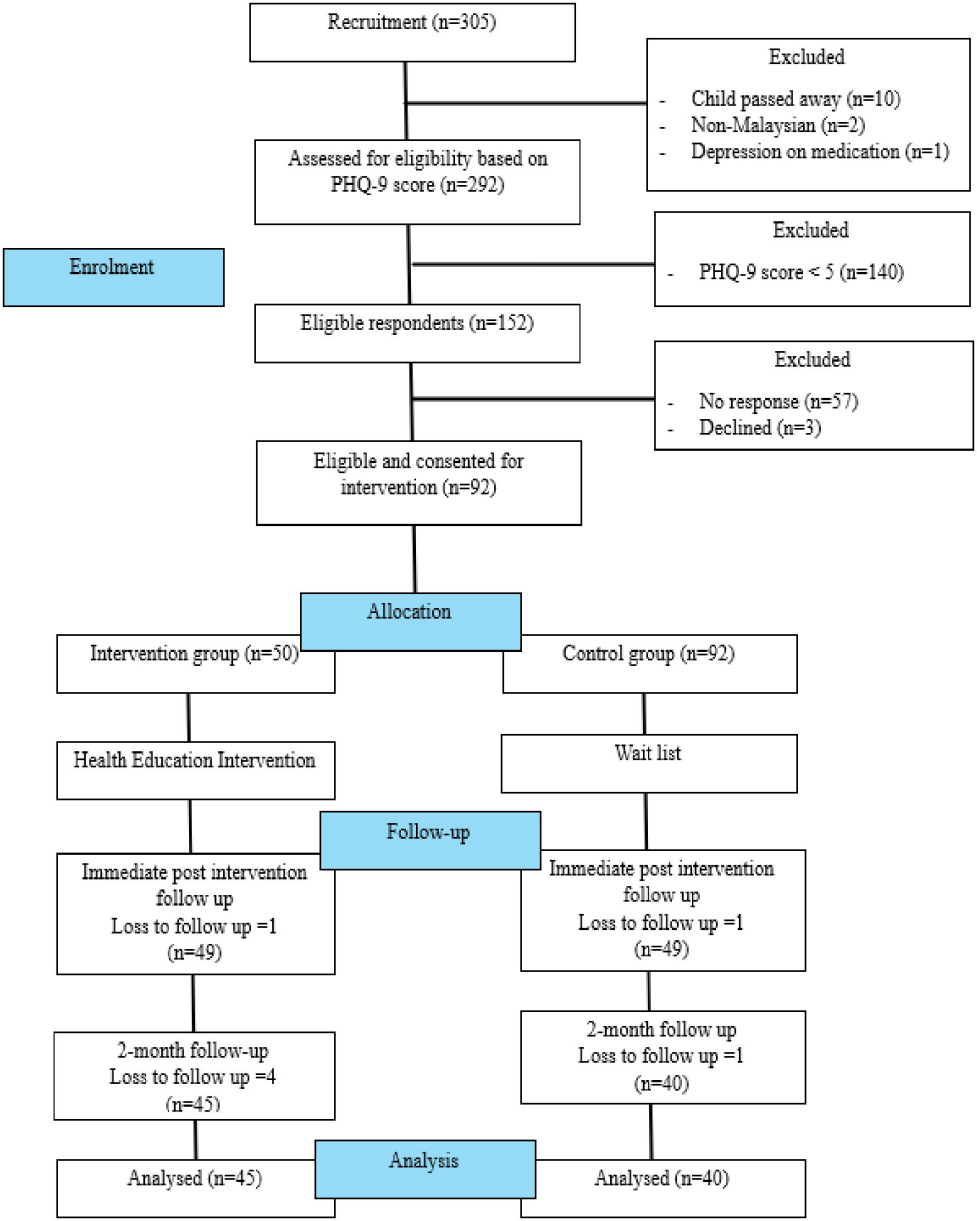
Flow of study participants.

A total of 305 mothers of children with cancers were assessed for eligibility. Of these, 13 mothers were excluded, including 10 mothers who had experienced the loss of their cancer child, 2 non-Malaysian mothers, and 1 mother who was taking anti-depressants. The remaining 292 mothers were screened for depression via the PHQ-9 questionnaire. Of 292 mothers, 152 scored ≥ 5 from PHQ-9 screening and were eligible for participation. These 152 mothers were invited to participate in the study; however, 57 mothers not responded, and 3 mothers refused to participate. Thus, only 92 mothers participated, with a response rate of 60.53% (92/152). Among these 92 respondents, 50 were from the intervention group and 42 from the control group. At the time of data collection immediately post-intervention (T2), 1 respondent from the intervention group and 1 respondent from the control group did not respond to the questionnaire. In subsequent follow-up (T3), 4 respondents from the intervention group and 1 from the control group did not respond to the questionnaires, giving rise to a 7.60% attrition rate. Fig 1 illustrates the flow of study participants. Moreover, this study applied intention-to-treat analysis, wherein all missing or incomplete data, whether from attrition or withdrawal were analyzed. The missing data were analyzed, giving rise to 7.60% missing data. The missing data came from dropped-out cases at T2 and T3. The missing value analysis by Little’s test showed that the data were missing completely at random (MCAR), x2 = 46.908, p = 0.086. These missing values were handled using Expectation Maximisation (EM) methods, computed via SPSS 25, which is appropriate when data are missing completely at random (MCAR), as confirmed by Little’s MCAR test. The EM method was chosen for its ability to provide unbiased estimates while maintaining data variability, particularly in studies with small sample sizes [36, 37].

#### Sociodemographic, depression scores, cancer knowledge scores, perceived stress scores, self-efficacy scores, coping scores, and social support scores between the intervention and control groups at baseline (N = 92)

Table 4 demonstrates no significant differences in sociodemographic characteristics between the intervention and control groups at baseline. Table 5 demonstrates no significant differences in depression scores, cancer knowledge scores, perceived stress scores, self-efficacy scores, coping scores, and social support scores between the intervention and control groups at baseline.

**Table 4:** Comparison of sociodemographic characteristics between intervention and control groups at baseline (n = 92).

Characteristics	Groups n (%)		U^b^/X^2^^c^	p-value
	Intervention	Control		
	n = 50	n = 42		
**Mothers’ characteristics**				
**Mother’s age**	37.00 (10.0)^a^	38.00 (9.3)^a^	-1.34^b^	0.18
**Ethnicity**				
Malay	43 (86.0)	40 (95.2)		0.17^d^
Non-Malay	7 (14.0)	2 (4.8)		
**Marital status**				
Married	48 (96.0)	39 (92.9)		0.66^d^
Single/divorced	2 (4.0)	3 (7.1)		
**Education**				
High school or less	19 (38.0)	16 (38.1)	0.00^c^	0.99
Diploma or higher	31 (62.0)	26 (61.9)		
**Employment**				
Employed	27(54.0)	24 (57.1)	0.09^c^	0.76
Housewife	23 (46.0)	18 (42.9)		
**Number of children**				
≤ 3	34(68.0)	26 (61.9)	0.37^c^	0.54
> 3	16 (32.0)	16 (38.1)		
**Household income**	5000 (3825)^a^	4000 (6250)^a^	0.68^b^	0.49
**Children characteristics**				
**Child’s age**	8.50 (7.0)^a^	9.00 (7.3)^a^	-0.59^b^	0.56
**Sex**				
Male	34 (68.0)	26 (61.9)	0.37^c^	0.66
Female	16 (32.0)	16 (38.1)		
**Cancer diagnosis**				
Haematological cancer	37 (74.0)	23 (54.8)	3.72^c^	0.05
Others	13 (26.0)	19 (45.2)		
**Duration since diagnosis**	3.50 (5.0)^a^	3.00 (5.0)^a^	0.226	0.82
**Treatment status**				
Active treatment	29 (58.0)	26 (61.9)	0.14^c^	0.70
Others	21 (42.0)	16 (38.1)		

^a^ Median (IQR-Interquartile range)

^b^ Mann Whitney U Test

^c^ Chi-Square test

^d^ Fisher Exact Test

*Significant at p-value < 0.05

**Table 5:** Baseline comparison between depression scores, cancer-knowledge scores, perceived stress scores, caretaker self-efficacy scores, problem-focused coping scores, emotion-focused coping scores, and social support scores between the intervention and control groups (N = 92).

Scores	Groups		U^a^	t^b^	p-value
	Intervention	Control			
**Depression scores**	7.0 (4.0)	8.0 (6.0)	-0.59		0.56
Median (IQR)					
**Cancer knowledge scores**	8.0 (2.0)	8.0 (2.0)	-0.98		0.33
Median (IQR)					
**Perceived stress scores**	15.1 ± 5.0	14.4 ± 6.1		0.68	0.24
Mean ± SD					
**Caretaker self-efficacy scores**	41.5 (20.0)	42.0(18.0)	-0.39		0.70
Median (IQR)					
**Coping (Problem-focussed)**	30.0 (8.0)	29.5 (6.0)	-0.57		0.57
Median (IQR)					
**Coping (Emotion-focussed)**	13.0 (4.0)	13.0 (3.0)	-0.71		0.48
Median (IQR)					
**Social support**	70.0 (18.0)	63.0 (20.0)	-0.55		0.45
Median (IQR)					

^a^: Mann Whitney U test

^b^: Independent t-test

Note: (*) Significant at p-value < 0.05

#### Effectiveness of SCODESS intervention on depression scores among the respondents

The effects of SCODESS intervention on depression scores were analyzed using Generalised Estimating Equations (GEE). Table 6 shows no significant interaction of depression scores between groups and time points. Specifically, there were no significant effects in the intervention group at immediate post-intervention compared to the control group at baseline (*β* = 0.70, 95% CI -2.41, 2.41, p-value = 0.909). Similarly, no significant effects in the intervention group at the 2-month follow-up compared to the control group at baseline (*β* = 1.26, 95% CI 3.09, 0.24, p-value = <0.622).

**Table 6:** Effects of SCODESS intervention on depression scores among mothers of children with cancer using GEE (n = 92).

Variables	β^a^	SE	Wald	95% CI		p-value
				Lower	Upper	
**Trial group**						
Control	Ref					
Intervention	-0.17	0.99	0.03	-2.12	1.79	0.88
**Timepoint**						
Baseline	Ref					
Immediate follow-up	-2.83	0.87	10.60	-4.54	-1.13	0.001*
2-month follow-up	-4.00	0.97	16.94	-5.90	-2.09	<0.001*
**Trial group x time point**						
**Control x baseline**	**Ref**					
**Intervention x immediate follow-up**	**0.13**	**1.16**	**0.01**	**-2.41**	**2.41**	**0.91**
**Intervention x 2 months follow-up**	**0.62**	**1.26**	**0.24**	**3.09**	**0.24**	**0.62**

^a^Intercept B coefficient 9.00,

*significant at p-value < 0.05

β = beta coefficient, SE = standard error, CI = Confidence Interval, Ref = Reference category

QIC = 6919.12, QICC = 6919.28

#### Effects of SCODESS intervention on cancer-related knowledge scores among the respondents

Table 7 shows the effects of SCODESS intervention on cancer-related knowledge scores among mothers of children with cancer using GEE. The intervention group had a significant 0.64 higher cancer-related knowledge scores immediately post-intervention compared to the control group at baseline (*β* = 0.64, 95% CI 0.22, 1.05, p-value = 0.002). At the 2-month follow-up, the intervention group had a significant 1.17 higher cancer-related knowledge scores compared to the control group at baseline (*β* = 1.17, 95% CI 0.65, 1.70, p-value = <0.001).

**Table 7:** Effects of SCODESS intervention on cancer-related knowledge scores among mothers of children with cancer using GEE (n = 92).

Variables	β^a^	SE	Wald	95% CI		p- value
				Lower	Upper	
**Trial group**						
Control	Ref					
Intervention	0.29	0.31	0.93	-0.30	0.89	0.33
**Timepoint**						
Baseline	Ref					
Immediate follow-up	0.02	0.13	0.03	-0.24	0.29	0.86
2-month follow-up	-0.60	0.20	8.91	-0.99	-0.20	**0.003** *
**Trial group x time point**						
**Control x baseline**	Ref					
**Intervention x immediate follow-up**	0.64	0.21	9.20	0.22	1.00	**0.002** *
**Intervention x 2 months follow-up**	1.17	0.27	19.23	0.65	1.70	**<0.001** *

^a^Intercept B coefficient 8.02,

*significant at p-value < 0.05

β = beta coefficient, SE = standard error, CI = Confidence Interval, Ref = Reference category

QIC = 551.76, QICC = 551.53

#### Effects of SCODESS intervention on coping skills among the respondents

Table 8 shows the effects of SCODESS intervention on coping skills (problem-focused and emotion-focused) among the respondents. There were significant effects of SCODESS intervention on problem-focused coping scores at immediate post-intervention (*β* = 2.50, 95% CI 0.42, 4.58, p-value = 0.018) and at the 2-month follow-up compared to the control group at baseline (*β* = 2.42, 95% CI 0.13, 4.72, p-value = 0.038). The intervention group had 2.50 higher problem-focused scores immediately post-intervention and 2.42 higher problem-focused scores at the 2-month follow-up compared to the control group at baseline.

**Table 8:** Effects of SCODESS intervention on coping skills scores (Problem-focussed and emotion-focussed) among mothers of children with cancer using GEE (n = 92).

Variables	β	SE	Wald	95% CI		p-value
				Lower	Upper	
**Problem-focused**						
**Trial group**						
Control	Ref					
Intervention	0.53	0.99	0.28	-1.42	2.47	0.61
**Timepoint**						
Baseline	Ref					
Immediate follow-up	-1.24	0.95	1.71	-3.10	0.62	0.19
2-month follow-up	-1.52	0.97	2.46	-3.43	0.38	0.12
**Trial group x time point**						
**Control x baseline**	Ref					
**Intervention x immediate follow-up**	2.50	1.10	5.56	0.42	4.58	**0.02** *
**Intervention x 2 months follow-up**	2.42	1.17	4.28	0.13	4.72	**0.04** *
**Emotion focussed**						
**Trial group**						
Control	Ref					
Intervention	0.31	0.56	0.32	-0.78	1.41	0.57
**Timepoint**						
Baseline	Ref					
Immediate follow-up	-0.36	0.51	0.50	-1.35	0.63	0.48
2-month follow-up	-0.69	0.49	1.99	-1.65	0.27	0.16
**Trial group x time point**						
**Control x baseline**	Ref					
**Intervention x immediate follow-up**	0.84	0.61	1.86	-0.37	2.04	0.17
**Intervention x 2 months follow-up**	1.15	0.68	2.87	-0.18	2.48	0.09

*Significant at p-value ≤ .05

*β = β coefficient*, SE = Standard Error, CI = Confidence Interval, Ref = Reference group

Problem focussed: Intercept B coefficient 29.10

Emotion focussed: Intercept B coefficient 13.17

However, no significant effects of SCODESS intervention were observed in emotion-focused coping; immediately post-intervention (*β* = 0.84, 95% CI -0.37, 2.04, p-value = 0.173) and at the 2-month follow-up (*β* = 1.15, 95% CI -0.18, 2.48, p-value = 0.090) compared to the control group at baseline.

#### Effects of SCODESS Intervention on perceived stress scores, caretaker self-efficacy scores, and social supports scores among the respondents

Table 9 shows no significant effects of SCODESS intervention on perceived stress scores, caretaker self-efficacy scores, and social support scores in the intervention group compared to the control group at baseline.

**Table 9:** Effects of SCODESS intervention on perceived stress scores, caretaker self-efficacy scores, and social support scores among mothers of children with cancer using GEE (n = 92).

Variables	β^a^	SE	Wald	95% CI		p-value
				Lower	Upper	
**Perceived stress scores**						
Control x baseline	Ref					
Intervention x immediate follow-up	0.29	0.10	0.09	-1.67	2.25	0.77
Intervention x 2 months follow-up	0.40	1.08	0.13	-1.73	2.52	0.72
**Caretaker self-efficacy scores**						
Control x baseline	Ref					
Intervention x immediate follow-up	-1.52	2.56	0.34	-6.61	3.58	0.56
Intervention x 2 months follow-up	4.19	2.61	2.59	-0.91	9.30	0.11
**Social supports scores**						
Control x baseline	Ref					
Intervention x immediate follow-up	2.90	2.23	1.68	-1.48	7.27	0.19
Intervention x 2 months follow-up	1.22	2.18	0.31	-3.05	5.50	0.58

*Significant at p-value ≤ .05

*β = β coefficient*, SE = Standard Error, CI = Confidence Interval, Ref = Reference group

Perceived stress: Intercept B coefficient 14.36, QIC = 9970.53, QICC = 9965.43

Caretaker self-efficacy: Intercept B coefficient 41.76, QIC = 21183.53, QICC = 21183.00

Social support: Intercept B coefficient 41.76, QIC = 60889.90, QICC = 60890.06

## Discussion

This study is the first to assess whether a brief online SCODESS intervention could improve depression scores, cancer knowledge scores, perceived stress scores, caretaker self-efficacy scores, coping skills scores, and social support scores among mothers of children with cancer at University Hospitals in Klang Valley, Malaysia.

The findings of this study showed no significant effects of the SCODESS intervention on depression scores among the respondents. Previous health education intervention studies on depression have yielded mixed results. Some studies reported significant improvements in depression scores [38–40] while a Randomised Controlled Trial by Marsland et al. (2020) involving 120 mothers found no significant effect on depression (p = 0.11) [41]. A meta-analysis of RCTs on interventions for family caregivers of cancer patients also showed no significant reduction in depression [23].

Several factors could explain these findings. Firstly, the variation in the duration of the intervention could significantly influence the outcomes. The one-week duration of intervention in this study might have been insufficient to produce a significant reduction in depression. Previous studies have implemented interventions lasting from two to six months, demonstrating more relevant effects on depression. However, although Marsland et. al, 2020 [41] conducted an eight-week intervention, there was also no significant improvement in depression among the mothers. One of the feedback received from the participants was that they felt the program was too short, and it ended while they were still confronting multiple cancer-related stressors [41]. Therefore, a longer duration of intervention is needed to address the complexity of depression and to observe meaningful improvements in symptoms Secondly, the depth of the intervention also matters. Studies involving direct supervision by psychologists or therapists have shown significant reductions in depression scores [39, 40]. The SCODESS intervention, delivered online through health education videos, lacked this level of engagement, which might have contributed to its limited effectiveness.

Thirdly, cultural factors, including mental health stigma, could significantly impact intervention outcomes. Higher stigma in Eastern countries like Malaysia might reduce the likelihood of seeking treatment and affect the acceptance of depression interventions [42–44]. Future interventions should address cultural barriers to improve engagement and effectiveness. Fourthly, the influences of external factors that were not controlled in this study might contribute to this outcome. The nature of depression is complex and influenced by various factors. Its natural course can fluctuate due to life changes, such as relationship issues, job loss, or even deterioration of the child’s health status. For instance, a respondent with a child recently diagnosed with relapse could be more depressed compared to a respondent whose child responded well to treatment. Furthermore, receiving assistance or varying levels of social support outside the intervention might also influence the depression outcomes. Therefore, future research should consider external factors and life events that could influence depression scores, thus helping to isolate the effects of the intervention more precisely.

Finally, another potential reason for the lack of significant findings could be the provision of a poster on depression to the control group. This poster, which included information on the definition, signs and symptoms, management of depression, and hotline numbers, might have inadvertently influenced the control group’s depression scores. While the intention was to provide basic mental health resources, the information in the poster might have served as a minimal intervention in itself, leading to a reduction in depressive symptoms in the control group. This information could have narrowed the gap between the intervention and control groups, thus reducing the observable impact of the SCODESS intervention. Future studies should carefully consider the type and amount of information provided to control groups to ensure it does not confound the intervention result manage these conditions. While subgroup analyses based on baseline depression levels were not conducted, it was acknowledged that such analyses could provide valuable insights into the effectiveness of the intervention in specific subgroups, and recommend this as a direction for future research.

Although there was no improvement in depression scores, significant improvement was observed in the cancer knowledge scores. The SCODESS intervention significantly increased cancer knowledge at immediate follow-up (p = 0.002), and at 2-month follow-up (<0.001) compared to the control group at baseline. This finding aligns with an educational intervention study conducted in another University Teaching Hospital in Malaysia, which also showed significant improvement in cancer knowledge post-intervention (p<0.01) [22].

The previous study by Othman et al. (2010) [22], utilized face-to-face intervention delivery, the current SCODESS study used an online method. Despite this difference, both studies demonstrated significant improvements in cancer knowledge. This consistency can be attributed to technological advancements, with many mothers today preferring online interventions. Furthermore, the effectiveness of the SCODESS intervention in improving cancer-related knowledge scores among the mothers is partly because the cancer knowledge content in the SCODESS intervention was designed to be relevant to the respondents. By tailoring the information to address the needs and concerns of mothers, more impactful interventions could be offered. Additionally, clear, and concise educational content improved knowledge retention and understanding. The repetition and reinforcement of key information over one week helped solidify the knowledge gained among the respondents.

Another key finding from this study was that the SCODESS intervention led to a 2.50 unit higher in problem-focused coping scores immediately post-intervention (p value = 0.018) and a 2.42 unit higher at the 2-month follow-up (p = 0.038) compared to the control group at baseline. However, no significant effects were observed on emotion-focused coping immediately post-intervention (p = 0.173) or at the 2-month follow-up (p = 0.090). Emotion-focused coping was based on indirect ways of problem-solving whereas problem-focused coping was based on direct ways of problem-solving [45]. In other words, emotion-focused coping involves managing emotional responses, while problem-focused coping involves actively mitigating the situation’s severity.

Comparatively, a study in India demonstrated that an intervention significantly improved active coping (p = 0.033) and reduced maladaptive coping, such as denial (p < 0.001), self-blame (p < 0.001), venting (p < 0.001), and behavioral disengagement (p < 0.001) [46]. This aligns with the SCODESS intervention’s improvement in problem-focused coping; but not emotion-focused coping. Similarly, an Egyptian study with caregivers of children with cancer showed significant improvement in coping skills immediately post-intervention (p < 0.001) and at 1-month post-intervention (p < 0.001) [47]. This intervention involved twice-weekly health education and group discussions over six weeks, using the Coping Health Inventory for Caregiver Parents (CHIP) as the coping measurement tool. Differences in intervention duration and measurement tools might account for the discrepancy in findings compared to the SCODESS study.

In conclusion, while the SCODESS intervention shows promise in enhancing problem-focused coping, there is a need for more comprehensive and prolonged interventions, such as longitudinal studies, to assess the sustainability of these effects. Additionally, coping can be categorized in various ways, as seen in previous studies [45–47]. Further exploration and elaboration are needed to understand coping mechanisms among these vulnerable mothers.

The study, however, did not demonstrate any significant effects of SCODESS intervention on other secondary outcomes (perceived stress, caretaker self-efficacy, and social support scores). This contrasts with a study by Marsland et. al. (2020) who showed significant stress improvement with a more extensive 12-session intervention [41]. As for caretaker self-efficacy and social support, respondents at baseline already had high self-efficacy and social support scores compared to other studies [21, 46]. Thus, possibly limiting the detection of changes over time for these measures. Given the high baseline scores, future interventions should be designed to address specific needs in caretaker self-efficacy and social support to detect significant changes over time.

### Strengths and limitations

The SCODESS module was developed based on an established theoretical model, Social Cognitive Theory (SCT). This study provides valuable evidence on the application of SCT model intervention in reducing depression among mothers of children with cancer. It evaluated multiple outcomes, including depression, cancer-related knowledge, caretaker self-efficacy, perceived stress, coping skills, and social support, providing a comprehensive assessment of the intervention’s impact.

Furthermore, one of the strengths of the study was the online delivery of the SCODESS intervention. Despite some limitations of online intervention, such as lack of personalized touch, less clarification on the module content, and potential poor adherence, the online delivery offers practicality and convenience for mothers of children with cancer. It allowed mothers to access health education materials at their convenience, fitting them into their caregiving responsibilities without the need for additional support. This aspect is crucial as it helps to overcome barriers like travel time, distance, and cost, thus making the intervention more accessible to a wider range of mothers.

Furthermore, the SCODESS intervention is the first to address depression among mothers of children with cancer at University Hospitals in Klang Valley, Malaysia. While it did not show a significant interaction between time and group for depression, it significantly improved cancer-related knowledge and problem-focused coping. These findings can serve as a baseline for future research in this area.

There are several limitations to this study. First, due to the voluntary nature of participation, there may be some selection bias among those who volunteered. Mothers who chose to participate may represent a proportion of individuals in need of help and who are open to online-based interventions. This may result in outcomes that differ from the general population. Secondly, the quasi-experimental design lacks the randomization of a Randomized Controlled Trial, potentially leading to selection bias. While it allows for comparisons between the intervention and control groups, the lack of randomization means differences may not be solely attributable to the intervention. Thirdly, the small sample size with specific study locations (University Hospitals) may limit the generalizability of the findings to a broader population of mothers caring for children with cancer. Fourthly, depression as the primary outcome is subject to fluctuate over time due to life circumstances. For example, if a child’s health deteriorated during the intervention, the mother’s depression scores could increase. Conversely, good news about the child’s recovery could lower the depression scores, even without intervention. Future research should consider life changes as potential confounders affecting the outcomes. Finally, the SCODESS intervention is an online health education intervention. While some respondents actively engaged, others did not. It was challenging to verify if everyone watched all videos to completion, particularly among less responsive respondents. Everyone was encouraged to be honest about their participation. Researchers adopted a non-judgemental attitude, assumed that all respondents watched all the health education videos provided.

## Conclusion and recommendations

The SCODESS intervention did not significantly affect depression scores as the primary outcome, but it improved cancer-related knowledge scores and problem-focused coping scores at follow-ups. The short duration of the program (one week) and lack of personal interaction may have limited its ability to significantly reduce something as complex as depression which requires more time and support. Longitudinal studies are recommended to assess the long-term impact, particularly for depression, which requires sufficient time to show improvement. Additionally, the sustainability of positive outcomes such as cancer-related knowledge and problem-focused coping among the mothers should be re-assessed over an extended period.

As a potential preventive strategy for depression, the theoretical framework could be modified by extending other variables that influence depression among mothers of children diagnosed with cancer, which were not included in this study due to time and other constraints. Variables such as the child’s behavioral factors, recent life change events (e.g. deterioration of the child’s health), distance from the hospital, additional supports, availability of current policies, treatment cost, and legislation should be explored in future studies. Identifying these factors can uncover other potential influences on depression scores among these mothers.

To enhance the practical utility of the SCODESS intervention, future adaptations should consider implementing the program in non-hospital settings and through various mediums to broaden the target population. Community health centers or local support organizations could serve as alternative venues for intervention delivery. These familiar and accessible locations can help reduce barriers such as travel and time constraints, enabling more mothers to participate without the logistical challenges associated with hospital visits. For example, collaboration with childhood cancer NGOs or parent support groups could facilitate the delivery of SCODESS intervention to the mothers.

Additionally, leveraging technology-based solutions beyond traditional online videos could improve engagement and program effectiveness. Mobile health (mHealth) applications with user-friendly interfaces and interactive features can provide participants with personalized health education, mental health check-ins, and real-time feedback from mental health professionals. Features like reminders, progress tracking, and interactive quizzes embedded in the app could reinforce learning and engagement. Such an app should monitor user engagement and facilitate interactive discussions, providing a more engaging experience. Additionally, a structured system for monitoring two-way communication should be established, with clear protocols for handling distressing content or misinformation to ensure adherence to the study guidelines. Involving co-moderators or utilizing automated tools could further improve oversight and participant safety, addressing potential risks and increasing the intervention’s overall effectiveness. By making these changes, it is hoped that the intervention could be more effective in improving depression outcomes in the future.

## Supporting information

S1 Data (XLSX)

S2 Data (XLSX)
